# Docosahexaenoic Acid Signaling Modulates Cell Survival in Experimental Ischemic Stroke Penumbra and Initiates Long-Term Repair in Young and Aged Rats

**DOI:** 10.1371/journal.pone.0046151

**Published:** 2012-10-30

**Authors:** Tiffany N. Eady, Ludmila Belayev, Larissa Khoutorova, Kristal D. Atkins, Changde Zhang, Nicolas G. Bazan

**Affiliations:** Neuroscience Center of Excellence, Louisiana State University Health Sciences Center, New Orleans, Louisiana, United States of America; Julius-Maximilians-Universität Würzburg, Germany

## Abstract

**Background:**

Docosahexaenoic acid, a major omega-3 essential fatty acid family member, improves behavioral deficit and reduces infarct volume and edema after experimental focal cerebral ischemia. We hypothesize that DHA elicits neuroprotection by inducing AKT/p70S6K phosphorylation, which in turn leads to cell survival and protects against ischemic stroke in young and aged rats.

**Methods and Results:**

Rats underwent 2 h of middle cerebral artery occlusion (MCAo). DHA, neuroprotectin D1 (NPD1) or vehicle (saline) was administered 3 h after onset of stroke. Neurological function was evaluated on days 1, 2, 3, and 7. DHA treatment improved functional recovery and reduced cortical, subcortical and total infarct volumes 7 days after stroke. DHA also reduced microglia infiltration and increased the number of astrocytes and neurons when compared to vehicle on days 1 and 7. Increases in p473 AKT and p308 AKT phosphorylation/activation were observed in animals treated with DHA 4 h after MCAo. Activation of other members of the AKT signaling pathway were also observed in DHA treated animals including increases in pS6 at 4 h and pGSK at 24 h. DHA or NPD1 remarkably reduced total and cortical infarct in aged rats. Moreover, we show that in young and aged rats DHA treatment after MCAo potentiates NPD1 biosynthesis. The phosphorylation of p308 AKT or pGSK was not different between groups in aged rats. However, pS6 expression was increased with DHA or NPD1 treatment when compared to vehicle.

**Conclusions:**

We suggest that DHA induces cell survival, modulates the neuroinflammatory response and triggers long term restoration of synaptic circuits. Both DHA and NPD1 elicited remarkable protection in aged animals. Accordingly, activation of DHA signaling might provide benefits in the management of ischemic stroke both acutely as well as long term to limit ensuing disabilities.

## Introduction

Stroke is the fourth leading cause of death and the leading cause of adult disability in the US. Age is the most important independent risk factor for stroke [1]. The incidence, disability and mortality associated with stroke increases with age [2]. Although there have been many investigations into the consequences of stroke and neuroprotective strategies, very few studies have been performed on aged animals. As a result, over one thousand clinical trials based on promising preclinical data mostly collected from young animals have failed [3].

Focal cerebral ischemia produces an irreversibly injured core and a peripheral zone (penumbra) of damaged tissue that is potentially salvageable [4]. The core is an area of severe ischemia with local cerebral blood flow (lCBF) below 20 ml/100 c/min, where the oxygen and glucose shortage results in rapid depletion of energy stores. Severe ischemia results in necrosis of neurons and supporting glia within the core. The penumbra is a rim of mild to moderately ischemic tissue partially perfused (lCBF below 40 ml/100 c/min) and the area in which the infarction actively evolves. Unlike the core, the penumbra may remain viable for several hours because it is supplied with blood by collateral arteries with branches of the occluded vascular tree [4]. However, even cells in this region will die if reperfusion is not established during the early hours after ischemia, since collateral circulation is inadequate to sustain neuronal demand for nourishment. In addition to reduced lCBF as a major factor responsible for necrotic injury, other events, including lipid peroxidation, inflammatory responses and brain edema, contribute to the severity and/or progression of penumbral injury. As time elapses after the onset of stroke, the extent of the penumbra decreases and undergoes irreversible damage unless reperfusion is initiated [5].

Cerebral ischemia-reperfusion increases intracellular calcium and activates several degradative enyzmes including phospholipases A2 [6], resulting in the rapid accumulation of free (unesterified) docosahexaenoic acid (DHA; 22:6, n-3) and arachidonic acid (AA; 20:4 n-6). The pool size enhancement of these fatty acids leads to the initiation of oxygenation pathways towards the biosynthesis of eicosanoids and docosanoids [7]. Free radical mediated lipid peroxidation takes place during the reperfusion phase and becomes a trigger of cell damage [8]. DHA is converted to the bioactive mediator NPD1 (10R,17S-dihydroxy-docosa-4Z,7Z,11E,15E,19Z hexaenoic acid) through a pathway initiated by 15-lipoxygenase-1 (15-LOX-1) followed by epoxidation and hydrolysis reactions [9], [10], [11].

DHA is involved with brain and retinal development, aging, memory, synaptic membrane function, photoreceptor function, and neuroprotection [12], [13], [14], [15]. Treatment with DHA improves behavioral outcomes, reduces infarct volumes, and decreases mortality in focal cerebral ischemia in young rats when administered within five hours of stroke onset [16]. NPD1 also elicits neuroprotection during oxidative stress and exhibits potent inflammatory resolving bioactivity by inhibiting leukocyte infiltration, cyclooxygenase-2 expression, and interleukin 1β-induced NFκB activation [7]. NPD1 also counteracts apoptosis in retinal epithelial cells [9]. Moreover, this lipid mediator attenuates the consequences of oxidative stress induced by H_2_O_2_ and tumor necrosis factor by activating anti-apoptotic proteins Bcl-2 and Bcl-xL, while inhibiting pro-apoptotic proteins BAD, BAX and Caspase-3 [11]. After ischemia-reperfusion injury, endogenous NPD1 production in the mouse brain peaks 8 h after the onset of MCAo and reduces stroke infarct volume at 42 h [7]. In that study, we identified the endogenous formation and reported the bioactivity of a 10, 15 5-docosatriene. Subsequently we suggested to name this lipid NPD1 [9]. Thus DHA, via its downstream mediator NPD1, activates cell survival signaling cascades, tipping the overall cellular fate towards survival and long-term repair.

DHA increases phosphatidylserine (PS) in neuronal cells and in turn translocates AKT from the cytosol to the membrane after ischemia; a required step for phosphorylation and activation of AKT [17], [18]. Here we have explored downstream AKT phosphorylation events. Mammalian Target of Rapamycin Complex 1 (mTORC1) is a complex that is activated by the PI3K pathway [19]. mTORC1 includes multiple components such as TOR1, Raptor, and mLST8. Although the individual functions of these components are not well understood, together they function as a Ser/Thr kinase that positively regulates protein synthesis, transcription, cellular growth, survival, metabolism and development via the downstream target S6 [20]. Specifically, AKT strongly inactivates the mTORC1 inhibitory TSC1 (hamartin)-TSC2 (tuberin) complex [21].

The goal of this work is to define mechanisms by which DHA re-establishes cell survival signaling cascades, and to investigate the potential neuroprotective role of DHA and its docosanoid derivative NPD1 in aging. We hypothesize that lipid signaling pathways are disrupted in the elderly, which results in a reduced capacity to form NPD1 and subsequent poor functional outcomes after brain injury. We used LC-MS/MS based-lipidomic analysis in conjunction with behavioral, Western blots using antibodies for phosphorylation sites of AKT pathway proteins, and immunostaining methods to expand our understanding of the mechanisms, responsible for DHA and NPD1 induced neuroprotection in young and aged rats.

## Results

### DHA restores neurological deficits, decreases infarct size and induces cell survival in the brain of young animals after stroke

Rectal and cranial (temporalis muscle) temperatures, arterial blood gases, and plasma glucose showed no significant differences among groups (**Table 1**). There were no adverse behavioral side effects observed after DHA or NPD1 administration.

**Table 1:** Physiological Variables.

		Young animals			Aged animals	
	Saline	DHA	NPD1	Saline	DHA	NPD1
	(n = 8)	(n = 7)	(n = 8)	(n = 10)	(n = 9)	(n = 8)
**Before MCAo (15 min)**						
Rectal temperature (°C)	36.6±0.1	36.7±0.1	36.7±0.1	37.0±0.0	36.8±0.2	37.1±0.1
Cranial temperature (°C)	36.4±0.1	36.8±0.1	36.5±0.1	37.0±0.0	36.6±0.2	37.1±0.2
pH	7.42±0.01	7.42±0.01	7.43±0.01	7.44±0.02	7.44±0.01	7.46±0.01
PO2, mm Hg	123±8	127±10	119±7	100±7	109±9	108±7
PCO2, mm Hg	39±1	39±1	39±01	38±1	39±1	38±01
Plasma glucose, mg/dL	135±5	139±10	157±10	239±17	246±32	211±10
Body weight (g)	302±7	294±14	302±8	578±13	575±9	542±8
**During MCAo (15 min)**						
Rectal temperature (°C)	37.3±0.2	36.5±0.02	36.6±0.1	38.0±0.0	37.7±0.2	37.4±0.2
Cranial temperature (°C)	36.5±0.1	36.7±0.1	36.4±0.1	37.0±0.0	37.1±0.1	36.9±0.1
pH	7.42±0.01	7.40±0.01	7.43±0.01	7.44±0.01	7.44±0.01	7.46±0.01
PO2, mm Hg	106±3	106±4	107±4	90±5	93±2	99±4
PCO2, mm Hg	40±1	40±1	39±1	39±1	40±1	39±1
Plasma glucose, mg/dL	133±7	135±6	142±9	207±9	231±29	169±7
**After treatment (15 min)**						
Rectal temperature (°C)	36.9±0.2	36.4±0.3	36.8±0.2	37.0±0.0	36.4±0.1	37.0±0.2
Cranial temperature (°C)	36.4±0.1	36.1±0.2	36.2±0.2	37.0±0.0	36.9±0.2	36.8±0.2
**After treatment (24 h)**						
Rectal temperature (°C)	37.8±0.2	38.4±0.4	37.4±0.2	37.0±0.0	37.4±0.2	37.8±0.2
Body weight (g)	272±9	269±11	280±7	544±11	553±7	521±9
**After treatment (48 h)**						
Rectal temperature (°C)	37.6±0.3	37.7±0.4	37.1±0.1	37.0±0.1	37.6±0.3	37.9±0.1
Body weight (g)	269±10	261±9	284±7	510±13	537±6	519±11
**After treatment (72 h)**						
Rectal temperature (°C)	37.5±0.4	37.5±0.3	37.1±0.1	37.0±0.1	37.6±0.3	37.7±0.1
Body weight (g)	269±14	263±13	293±6	514±13	528±7	508±11
**After treatment (7 days)**						
Rectal temperature (°C)	37.6±0.7	37.6±0.3	37.1±0.2	37.0±0.0	37.9±0.2	37.5±0.3
Body weight (g)	284±25	301±11	322±7	510±16	531±7	520±8

**Values are mean ± SEM.**

**MCAo, middle cerebral artery occlusion.**

**Figure 1:**
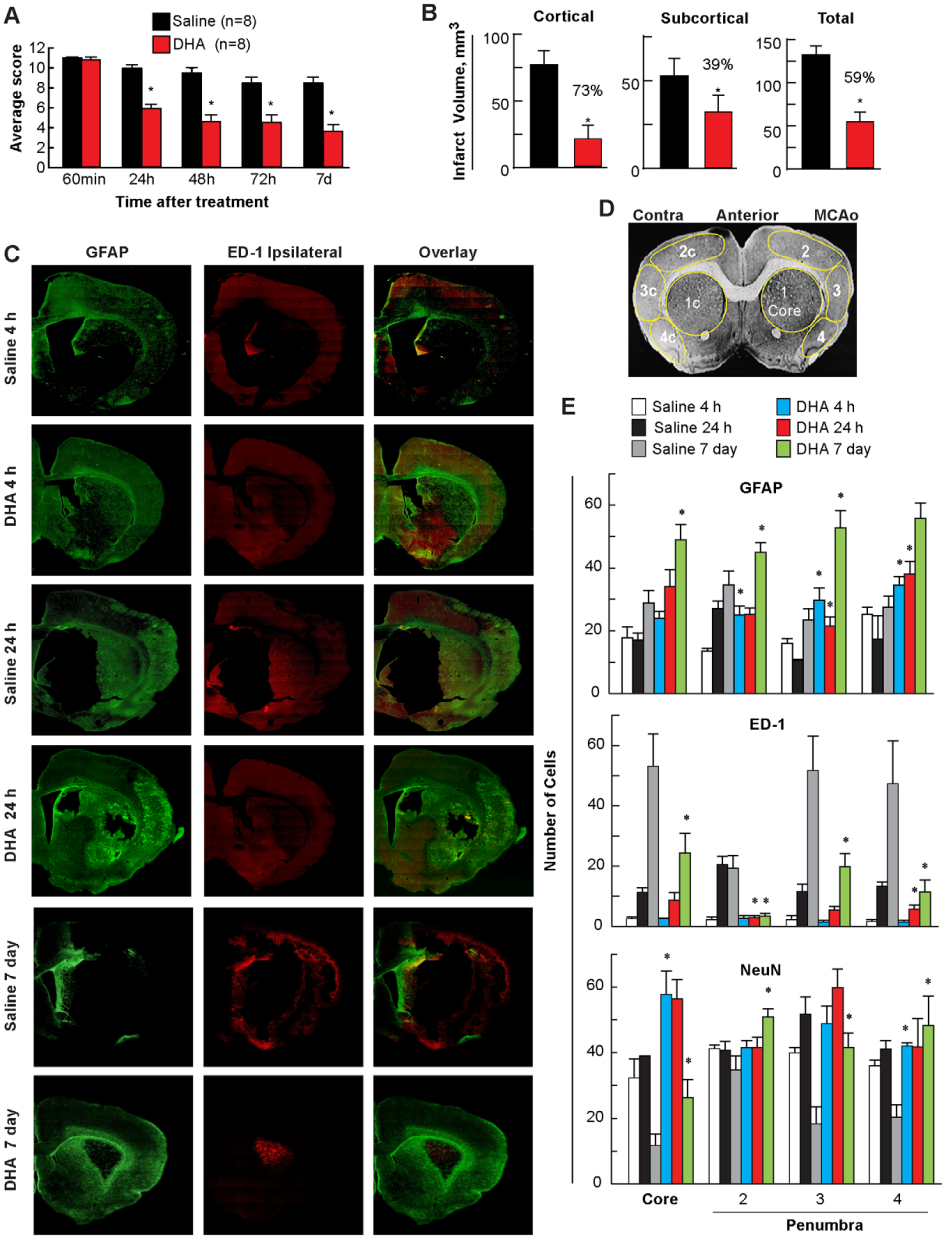
Behavioral, histopathology and immunohistochemistry in young rats. Panel A: Total neurological score at 24, 48, 72 h, and 7 days after MCAo. Panel B: Cortical, subcortical and total infarct volumes on day 7 after stroke. Panel C: Computer-generated MosaiX-processed images of GFAP (green), ED-1 (red), and GFAP/ED-1 double staining at 4 h, 24 h and 7 days after stroke at magnification ×10. Panel D is a schematic coronal brain diagram (bregma level +1.2 mm) showing locations of regions for cell counts in ischemic core (1) and penumbra (2, 3 and 4). Panel E: Number of GFAP-positive astrocytes, ED-1-positive microglia cells, and NeuN-positive neurons at 4, 24 h, and day 7 after onset of stroke. Data are mean ± SEM. *indicates significant differences from saline (p<0.05; repeated measures ANOVA followed by Bonferroni tests).

DHA administered at 3 h after MCAo onset improved behavioral scores at 24, 48, 72 h, and 7 days after MCAo (**Fig. 1A**) and reduced infarct volumes (**Fig. 1B**). Saline-treated rats showed extensive neuronal loss, GFAP-positive reactive astrocytes outlining the lesion territory, and massive ED-1-positive microglia/macrophage infiltration as early as 24 h (**Fig. 1C**). In contrast, DHA attenuated damage, increased GFAP-positive reactive astrocytes and decreased ED-1 positive microglia/microphages (**Fig. 1C**). Regions for GFAP, ED-1 and NeuN positive cell counts are presented in **Fig. 1**D. DHA increased GFAP (at 4 and 24 h, and 7 days), NeuN (at 7 days) and decreased ED-1 (at 24 h and 7 days) positive cell counts (**Fig. 1E**). These consequences of the DHA treatment, even at 7 days after the MCAo, provide the basis for the suggestion that a DHA- mediated long term repair cascade was set in motion.

### DHA on protein phosphorylation in the penumbra of young animals

**Figure 2:**
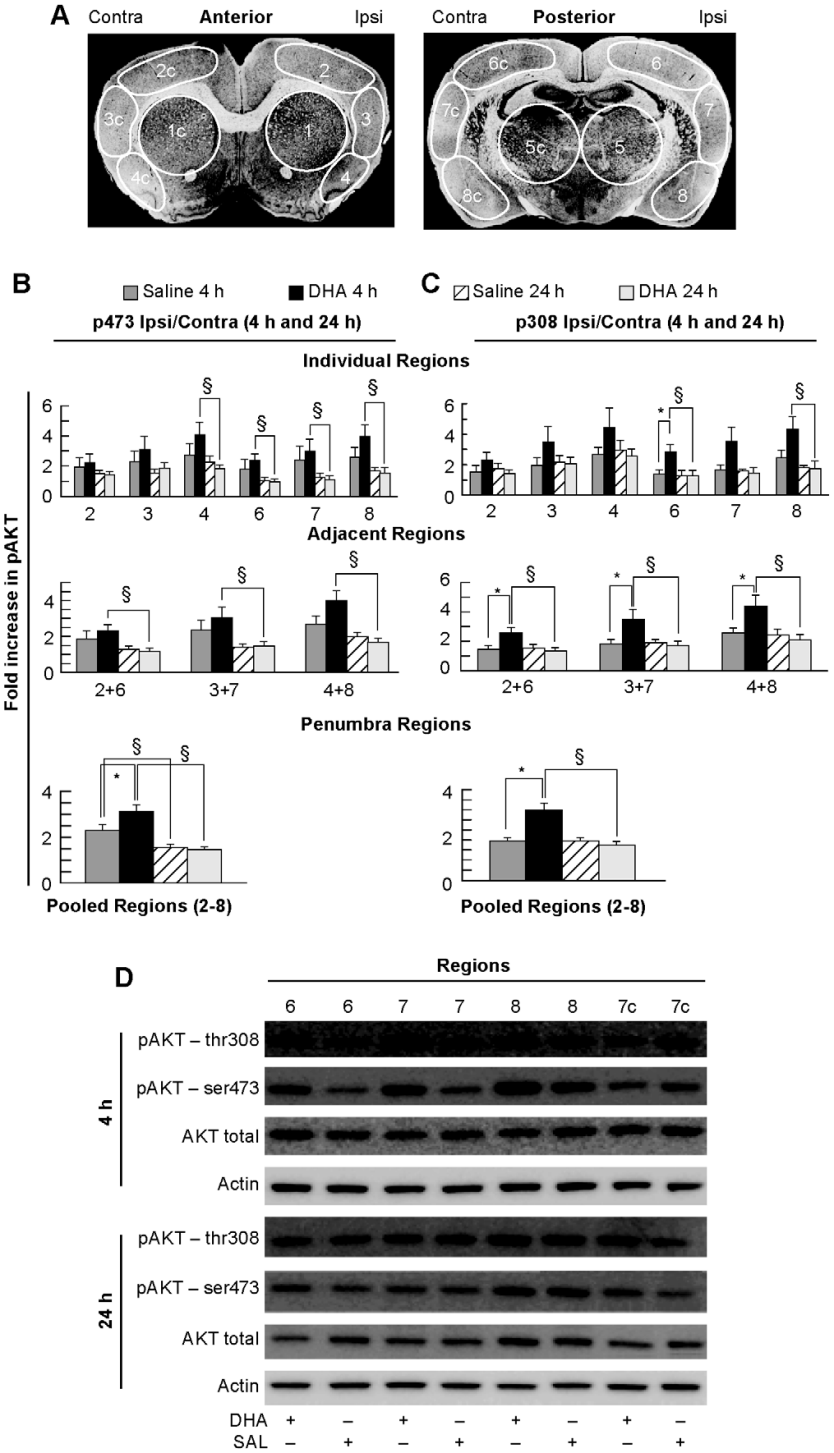
AKT phosphorylation in young animals. Panel A: Schematic of coronal brain diagrams (bregma levels +1.2 and −1.8 mm) showing locations of regions for western blot analysis, conducted at 4 and 24 h after onset of stroke. Individual regions of the penumbra (2, 3, 4 and 6, 7, 8) were analyzed separately and also pooled together for Panels B and C. Panel D: Representative Western blots of AKT trials showing increased AKT phosphorylation in DHA treated animals at 4 h. DHA significantly increased AKT phosphorylation at 4 h. Data are mean ± SEM. * and § indicate significant differences between groups (p<0.05; repeated measures ANOVA followed by Bonferroni tests).

The phosphorylation of AKT at serine 473 is a known consequence of ischemia reperfusion injury [22]. In order to examine the effect of DHA on this signaling, we investigated the phosphorylation status of AKT in the penumbral regions (**Fig. 2A**). When penumbral regions were analyzed, phosphorylation at p473 AKT was significantly up-regulated in DHA-treated animals at 4 h relative to 24 h (increased in region 4 by 122%, region 6 by 144%, region 7 by 168% and region 8 by 153%) (**Fig. 2B**). Phosphorylation at p473 was up-regulated in DHA-treated animals at 4 h relative to 24 h in all adjacent penumbral regions (region 2+6 by 94%, region 3+7 by 107%, region 4+8 by 138% increase). When all regions were pooled, phosphorylation at p473 was up-regulated in DHA-treated animals at 4 h relative to both saline-treated at 4 h (36% increase) and DHA-treated animals at 24 h (114% increase). In addition, saline-treated at 4 h had higher phosphorylation levels relative to saline-treated animals at 24 h (46% increase) consistent with previous reports on p473 AKT status after MCAo [22] (**Fig. 2B**). DHA-treated animals had increased phosphorylation of AKT at serine 473 above the levels of vehicle at 4 h with levels returning to baseline by 24 h in both DHA and vehicle-treated animals.

DHA-treated animals also had significantly up-regulated phosphorylation of AKT at threonine 308 at 4 h. Phosphorylation of p308 AKT was not altered in saline-treated animals (**Fig. 2C**). This suggests a correlation between DHA treatment and the selective activation of the p308 phosphorylation site after MCAo. When individual regions were analyzed, phosphorylation at p308 was up-regulated in the penumbra of DHA-treated animals at 4 h relative to 24 h (increased in region 6 by 121% and region 8 by 146%) and relative to saline-treated animals at 4 h (region 6 by 107% increase) (**Fig. 2C**). Phosphorylation at p308 was up-regulated in DHA-treated animals at 4 h relative to 24 h (regions 2+6 by 90%; regions 3+7 by 101% and regions 4+8 by 146%) and relative to saline-treated animals at 4 h (increased regions 2+6 by 78%; regions 3+7 by 91% and regions 4+8 by 69%) in all adjacent penumbral regions (**Fig. 2C**). When all regions were pooled, phosphorylation at p308 was up-regulated in DHA-treated animals at 4 h relative to both saline-treated at 4 h (79% increase) and DHA-treated animals at 24 h (98% increase). There was no difference in the phosphorylation state of saline-treated animals between 4 and 24 h (**Fig. 2C**). Representative Western blots showing the patterns of AKT activation in DHA-treated animals versus vehicle at 4 h and 24 h are presented (**Fig. 2D**).

**Figure 3:**
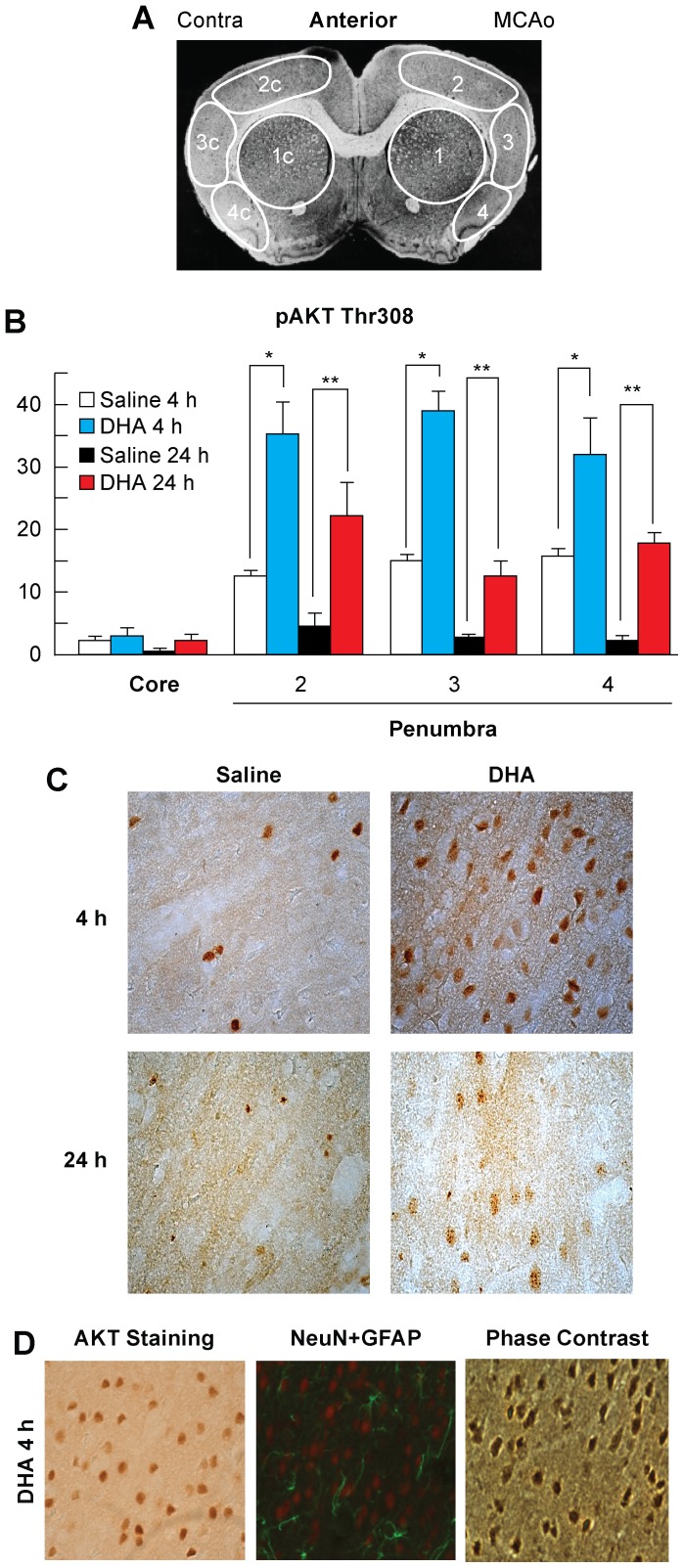
AKT p308 immunohistochemistry in young rats. Panel A:Schematic coronal brain diagram showing locations of regions for cell counts in core (1) and penumbra (2, 3 and 4). Panel B: Number of p308 AKT-positive cells at 4 and 24 h after MCAo. Panel C: Bright field images of p308 AKT-positive cells at 4 h and 24 h after 2 h of MCAo at a magnification ×40. Panel D: Evidence that p308 AKT stains neurons. Bright field image of AKT staining, computer-generated MosaiX-processed images of GFAP (green)/NeuN (red) double staining (overlay) and phase contrast image of DHA treated rat at 4 h after 2 h of MCAo at a magnification ×40 Data are mean ± SEM. * and ** indicate significant difference between groups (p<0.05; repeated measures ANOVA followed by Bonferroni tests).

To further extend these observations we conducted immunohistochemistry of p308 AKT (**Fig. 3A**). At 4 h, positive staining cells had a very opaque uniform staining. DHA-treated animals had higher cell counts of p308 AKT-positive cells in the penumbra at 4 h (region 2 by 182%, region 3 by 160% and region 4 by 103%) and also at 24 h in all regions as compared to saline-treated animals (**Fig. 3B**). Interestingly, cells stained 24 h after the ischemic insult, although developed at the same time and manner as at 4 h, had less uniform pigment with a much lighter, spotted staining pattern (**Fig. 3C**). Images of activated p308 AKT-positive cells were compared with NeuN-positive cells by phase microscopy. The punctate appearance of triangular cells tracking through the tissue in parallel paths suggests the visualized cells are neuronal somas (**Fig. 3D**).

**Figure 4:**
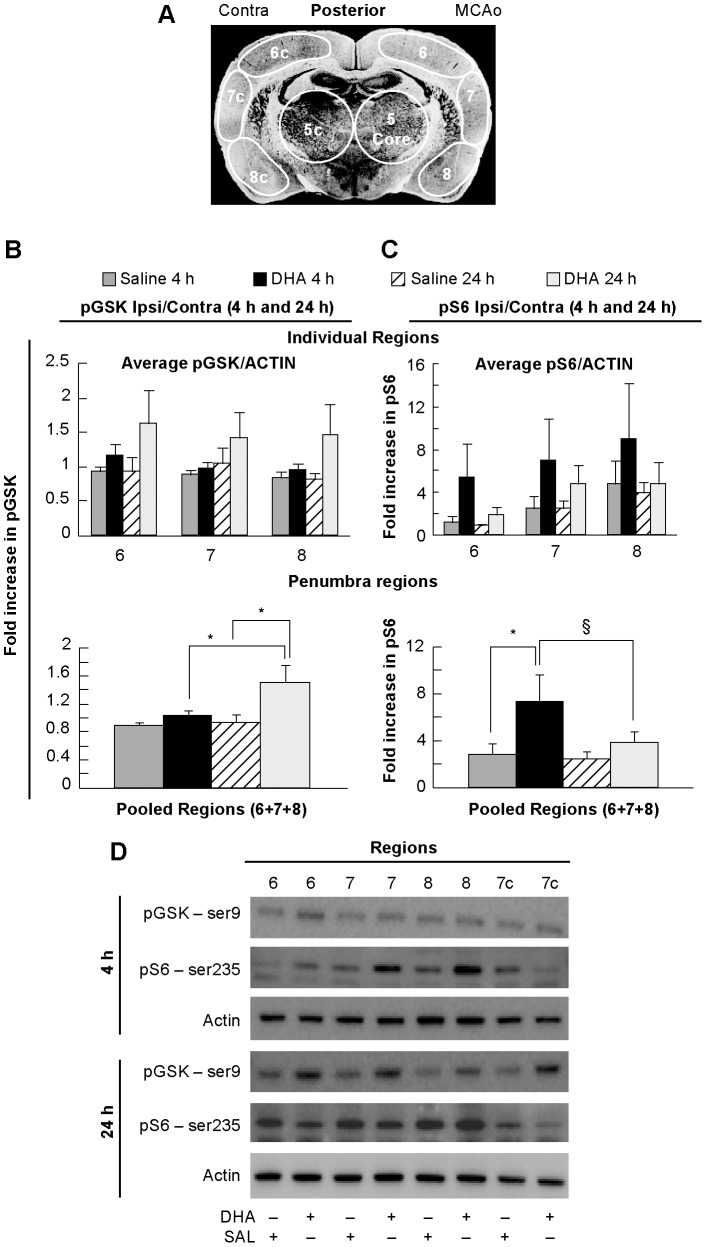
GSK and S6 phosphorylation in young rats. Panel A: Schematic coronal brain diagram (bregma level −1.8 mm) showing the locations of regions for western blot analysis, conducted at 4 and 24 h after onset of stroke. Individual regions of the penumbra or pooled penumbral regions were analyzed. Panel B: Treatment with DHA results in an increase of phosphorylation at pGSK (ser 9) at 24 h when compared to saline at 24 h and DHA at 4 h. Panel C: Treatment with DHA results in an increase of phosphorylation at pS6 (ser 235) when compared to Saline at 4 h. Panel D: Representative Western blots of GSK and S6 trials. Data are mean ± SEM. * and § indicate significant differences between groups (p<0.05; repeated measures ANOVA followed by Bonferroni tests).

AKT is part of a ubiquitous pathway of protein synthesis, cell survival, proliferation, glucose metabolism and neuronal homeostasis. We assessed AKT phosphorylation of downstream mediators in the penumbra after DHA treatment (see diagram in **Fig. 4A**). To uncover DHA mediated signaling we looked at GSK phosphorylation as a marker of AKT activity. We found that pGSK was elevated at 24 h in DHA-treated animals relative to DHA at 4 h by 45% and saline-treated animals at 24 h by 61% (**Fig. 4B**). Because both saline and DHA-treated animals have activated p473 AKT at 4 h, one would expect them to have similar pGSK levels. The elevated pGSK at 24 h specifically in DHA-treated animals suggests that DHA-treated animals also experience prolonged AKT activity out to at least 24 h in addition to increased levels of AKT phosphorylation levels at 4 h. Next we investigated the S6 phosphorylation status at 4 and 24 h. S6 is a ribosomal protein and phosphorylation indicates activated ribosomes and vigorous protein translation. We found that DHA-treated animals had increased pS6 levels over saline-treated animals at 4 h by 160% (**Fig. 4C**). The levels of pS6 were elevated at 24 h, but not enough to reach statistical significance. Representative Western blots of pGSK and pS6 are presented in **Fig. 4D**.

### DHA or NPD1 improves neurological deficit and reduces infarct size in aged animals

**Figure 5:**
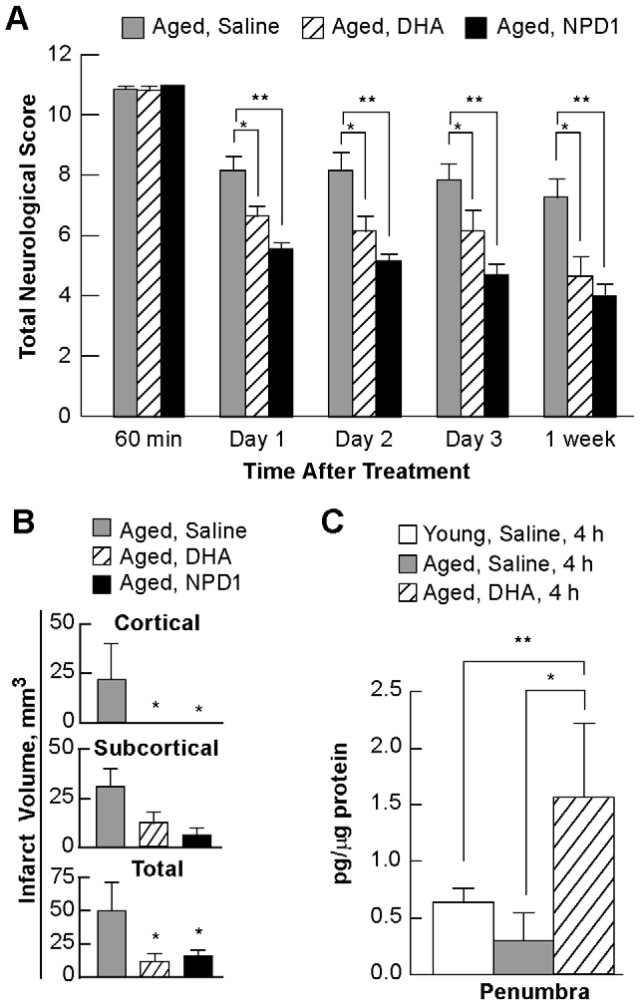
Behavioral, histopathology and lipidomic analysis in aged rats. Panel A: Behavioral outcomes over 7 days. Panel B: Total, cortical and subcortical infarct volumes on day 7 after stroke. Panel C: NPD1 content was analyzed by lipidomic analysis at 4 h in young and aged rats treated with either saline or DHA. Data are mean ± SEM. * and ** indicate significant difference between groups (p<0.05; repeated measures ANOVA followed by Bonferroni tests).

In order to investigate stroke susceptibility and poor recovery in aged individuals we examined the treatment response of aged rats (15–17 months) and quantified their neurobehavioral recovery after MCAo. DHA or NPD1 treatment resulted in improved neurobehavioral outcomes on days 1, 2, 3 and 7 (**Fig. 5A**). Total infarct volume corrected for brain swelling was reduced by DHA or NPD1 (75% and 87%, respectively) compared with saline-treatment (**Fig. 5B**). In addition, the cortical infarct was virtually eliminated by DHA or NPD1 and subcortical infarct was also significantly reduced (by 76% and 88%, respectively) compared with saline-treatment (**Fig. 5B**).

Next we studied the capacity for aged animals to synthesize NPD1 after MCAo. Aged rats treated with DHA had increased NPD1 synthesis when compared to both young and aged rats treated with vehicle (147% and 425% increase respectively) (**Fig. 5C**). That the synthesis of this lipid mediator was enhanced was ascertained by confirming the presence of 17-HDHA, the stable derivative of 175-Hp DHAm the NPD1 precursor (data not shown).

### The effect of DHA or NPD1 on protein phosphorylation in the penumbra in aged animals

**Figure 6:**
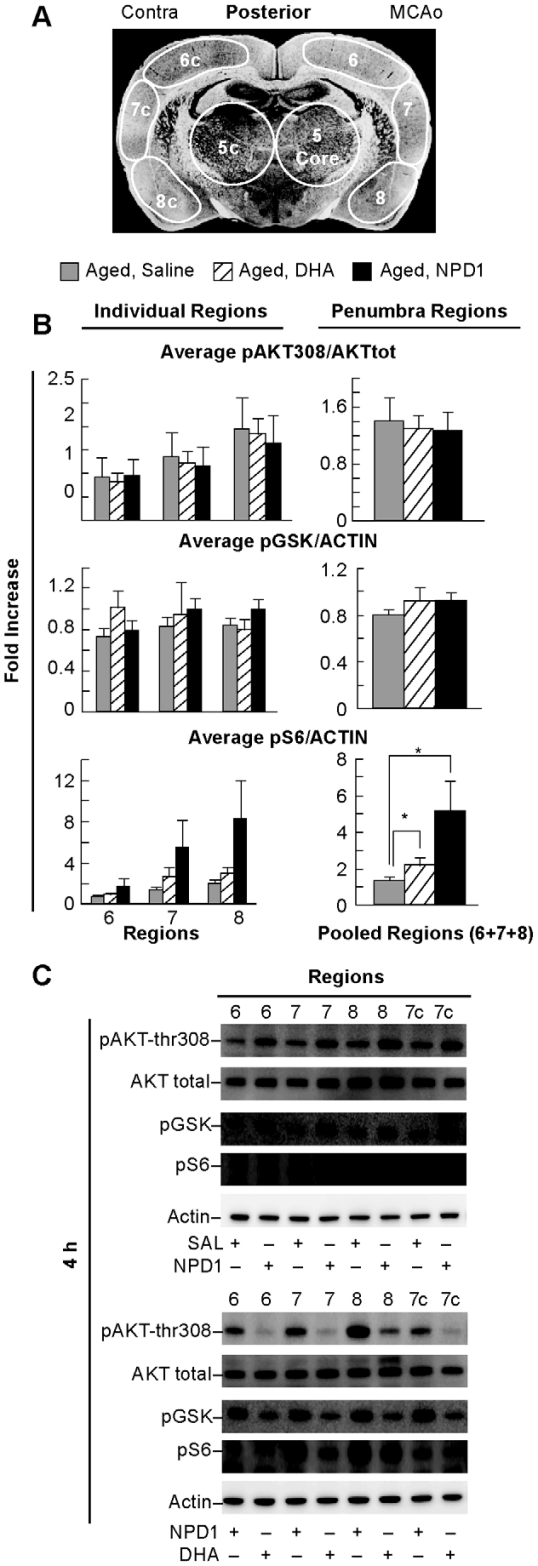
Protein phosphorylation in aged animals. Panel A: Schematic coronal brain diagram showing locations of regions of interest for Western blot analysis in the penumbra (regions 6, 7, and 8). Individual regions of the penumbra (6, 7, 8) were analyzed separately and also pooled together for Panel B. Treatment with NPD1 or DHA did not resulted in significantly different expression of phosphorylated p308 AKT or pGSK (ser9) over saline but resulted in higher levels of S6 (ser235) phosphorylation at 4 h when compared to saline-treated aged animals. Panel C: Representative Western blots of AKT p308, pGSK and pS6 trials in aged animals. Data are mean ± SEM. * indicates significant difference between groups (p<0.05; repeated measures ANOVA followed by Bonferroni tests).

We examined whether aged rats experience activation of AKT signaling after MCAo as young rats do. Young rats treated with DHA have increased phosphorylation of AKT at threonine 308 and pS6 at 4 h. To examine the effect of DHA or NPD1 on cell survival signaling in aged animals, we assessed phosphorylation status of AKT in the penumbral regions (see diagram in **Fig. 6A**) and observed no changes at 4 h on the phosphorylation state of p308 AKT or of pGSK (**Fig. 6B**). However, treatment with DHA or NPD1 corresponded with a significant effect on the phosphorylation/activation of pS6 in aged rats at 4 h when compared to vehicle. We show that DHA-treated animals had significantly increased pS6 levels over vehicle at 4 h by 62%, and NPD1 treated animals had an even higher increase of 280% (**Fig. 6B**). Representative Western blots are presented in **Figure 6C**.

## Discussion

Here we demonstrate that systemic administration of DHA after MCAo induces neurological recovery, reduces microglial infiltration, increases astrocytosis as early as 24 h and corresponds with the activation of AKT cascades as early as 4 h after the onset of ischemia. In addition, we show that aged rats synthesize NPD1 upon MCAo and this is event is potentiated by DHA treatment. Infusion of NPD1 is remarkably protective at 3 h after onset of stroke. Thus, the relative amount of endogenously produced NPD1 in response to severe ischemic insult (2 h after MCAo) might be insufficient to exert protection.

AKT pathways have been implicated in protein synthesis, cell survival, proliferation, glucose metabolism, injury-induced apoptosis, and neuronal maintenance. AKT achieves full activation when phosphorylated at both the Ser473 and Thr308 regulatory domains [23]. AKT phosphorylation results when phosphatidylinositol-4,5-bisphosphate (PIP2) is converted to phosphatidylinositol-3,4,5-triphosphate (PIP3) via phosphoinositide-3-kinase (PI3K). PIP3 activates phosphoinositide-dependent protein kinase (PDK1), which phosphorylates AKT's Thr308 domain [24]. PIP3 activity and AKT phosphorylation are often disrupted after stroke, resulting in a loss of AKT activity and increased cellular injury and apoptosis [25]. The domain at Ser473 is phosphorylated as a response to cellular stress via the rictor–mTORC1 [24]. Once activated, AKT phosphorylates mTORC1, Bcl-2-associated death promoter, forkhead transcription factor (FKHR), glycogen synthase kinase 3β (GSK3β), and proline-rich AKT substrate (PRAS), initiating cascades leading towards cell survival [26].

We demonstrated here that DHA-treated animals have increased AKT p473 phosphorylation at 4 h in the penumbra. Western blot analysis revealed the up-regulation of p473 AKT protein expression with DHA treatment at 4 h, and the return of p473 AKT to baseline in both DHA and saline-treated animals by 24 h. In addition, DHA-treated animals displayed selectively elevated AKT p308 at 4 h. Phosphorylation of p308 AKT was not altered in saline-treated animals. This finding was confirmed with immunohistochemistry of coronal brain sections showing that DHA-treated animals had elevated p308 AKT-positive cells at 4 h. This data suggests that DHA treatment has a selective effect on the p308 phosphorylation site achieved within 4 h which persists beyond 24 h after MCAo. Activated p308 AKT-positive cells with respect to NeuN-positive cells and neuronal images via phase contrast microscopy revealed that p308 AKT-positive cells stain darkly and uniformly at 4 h and have a punctate appearance of triangular cells tracking through the tissue in parallel paths, indicative of neuronal somas. This suggests that DHA may activate neuronal AKT signaling pathways, providing a possible explanation for the increased rates of neuronal survival in DHA-treated animals observed 7 days after stroke.

GSK phosphorylation is elevated at 24 h in DHA-treated animals. Because both saline and DHA treatments have activated p473 AKT at 4 h, one would expect them to have similar pGSK levels at 4 h. The elevated pGSK at 24 h in DHA-treated animals suggests that DHA is associated with prolonged AKT activity. We then investigated S6 phosphorylation status after DHA treatment and showed that DHA-treated animals had increased pS6 levels over saline animals at 4 h.

Aged individuals are more susceptible to stroke and have poorer recovery after brain injury. Epidemiology indicates that diets enriched with DHA are associated with reduced risk of cognitive impairment and slow the progression of dementia and Alzheimer's disease [27]. When rats are fed low-DHA diets for one or more generations, clear deficits in cognition are observed. Age-related defects in antioxidant systems result in reduced liver fatty acid desaturase capacity and an increase in lipid peroxidation that further reduces DHA levels [27]. As a result, aging is associated with decreased levels of DHA in the brain. Aged rats display larger infarct volumes, more severe behavioral deficits, increased cellular degeneration, and increased mortality after stroke [2], [28], [29]. In addition, aged rats after MCAo present enhanced disruption of the blood-brain barrier (BBB), a greater degree of neuronal damage, reduced functional recovery following focal cerebral ischemia [28], [29] and deregulated plasticity and repair [2], [30]. Thus, endogenous cell survival cascades involving lipid signaling are likely dysregulated in aged animals after stroke. NPD1 synthesis requires both a free pool of DHA and 15-LOX-1 [14]. Neurotrophins are NPD1 synthesis agonists [31] and their availability in stroke is limited.

Exogenous DHA or NPD1 exerts protection when administered after MCAo in aged rats. As an early endogenous response to ischemia-reperfusion injury, DHA is cleaved from phospholipids by phospholipase A2. Free DHA is then converted to form the stereospecific mediator NPD1 by 15-LOX-1 activity [31] followed by epoxidation and hydrolysis [13]. Here we show for the first time that aged rats have the capacity to synthesize NPD1, and that this synthesis is significantly potentiated by DHA treatment.

In aged animals 4 h after reperfusion phosphorylation of p308 AKT or pGSK were not changed. However, treatment was associated with a significant effect on the phosphorylation of S6 in aged rats at 4 h, as DHA or NPD1 treated animals had enhanced pS6 activation. This indicates that while AKT survival pathways may be delayed or disrupted in aging, the protein translational machinery is still capable of activation by 4 h, and the extent to which it is induced depends on treatment and correlates with improved neurobehavioral outcome after either DHA or NPD1. Whether redundant signaling is activated by the lipid mediators or not remains to be ascertained.

Our data shows that rats treated with DHA at 3 h after onset of MCAo had decreased ED-1-positive microglia infiltration and increased GFAP-positive astrocytosis as early as 24 h, and enhanced neuronal survival at 7 days when compared to saline-treated rats. These results indicate that DHA treatment protects the neurons and astrocytes. These two cells interdependently contribute to the maintenance and protection of neurons via the secretion of neurotrophic mediators. GFAP reactivity may be associated with neuronal resistance to ischemic damage. The contributions of astrocytes to normal brain physiology have been demonstrated by many reports [32], [33]. Astrocytes can release a number of growth factors for neurons [32]. Some of these, such as nerve growth factor, may stimulate the neuron as a whole as well as promote neurite growth. Thus, GFAP expression may be related to a possible protective effect on the adjacent neurons. Our data may be interpreted as evidence for the engagement of protective mechanisms in astrocytes and neurons within regions of the brain that remain viable following focal cerebral ischemia. In addition, DHA treatment attenuated the activation of microglia. After injury, phagocytic microglia engulf debris and infarcted cellular material and secrete pro-inflammatory factors to trigger further microglial migration and proliferation. Microglia activate proinflammatory cytokines IL-1α, IL-1β and TNF-α, which amplify the inflammatory response leading to chronic damaging inflammation. DHA attenuates microglial migration/activation shifting away from microglial-induced chronic inflammatory damage.

Here, we show that DHA protects the penumbra after stroke with concurrent unregulation of both p473 AKT and p308 AKT, and S6 and pGSK. This is in agreement with NPD1 bioactivity in retinal pigment epithelial (RPE) cells undergoing oxidative stress that results in higher levels of AKT, mTOR, and p70S6K phosphorylation and increased rates of all survival [34]. Inhibition of PI3K or mTOR/p70S6K by wortmannin and rapamycin, respectively, increased apoptosis and inhibited phosphorylation of Akt and p70S6K induced by oxidative stress [34]. These studies suggest the survival benefit observed from treatment with NPD1 or DHA is elicited, at least in part, via the activation of Akt and p70S6K pathways. In the present study we also demonstrated biosynthesis of NPD1 in the aged brain in response to experimental stroke. Moreover, we uncovered remarkable protective bioactivity of NPD1 administered to aged animals after stroke, suggesting that NPD1 endogenously generated either was insufficient or located in an unsuitable cellular site to elicit protective bioactivity. These observations contribute to expand the significance of DHA-NPD1 signaling for brain protection and long term repair after stroke, particularly during aging.

## Materials and Methods

All studies were approved by the Institutional Animal Care and Use Committees of the Louisiana State University Health Sciences Center. Young male Sprague-Dawley rats (287–385 g, 3–4months, Charles River Lab) or aged Sprague-Dawley rats (515–630 g, 15–17months, Harlan Labs) were fasted overnight but allowed free access to water. Anesthesia was induced with 3.5% isoflurane in a mixture of 70% nitrous oxide and 30% oxygen. Rats were orally intubated and mechanically ventilated. Temperature probes were inserted into the rectum and the left temporalis muscles to maintain temperatures at 36–37°C during surgical procedures. The right femoral artery and vein were catheterized for blood sampling for arterial gases, pH, and plasma glucose.

### Focal cerebral ischemia

The right middle cerebral artery (MCA) was temporarily occluded for 2 h by an intraluminal-filament coated with poly-L-lysine, as we previously described [35]. Briefly, the right common carotid artery (CCA) was exposed through a midline neck incision and dissected free of the surrounding nerves; the occipital branches of the external carotid artery (ECA) were coagulated and the pterygopalatine artery was ligated. A 4 cm length of 3-0 monofilament nylon suture was inserted via the proximal ECA into the internal carotid artery (ICA) and MCA a distance of 20–22 mm from the CCA bifurcation, according to the animal's weight. Following suture placement, the neck incision was closed, animals were allowed to awaken from anesthesia, and then they were tested on a standardized neurobehavioral battery. After 2 h of MCAo, rats were re-anesthetized with the same anesthetic combination. Temperature probes were reinserted, intraluminal sutures were carefully removed, and the animals were allowed to survive with free access to food and water according to the study protocol.

### Behavioral tests

Behavioral tests were performed by an observer blinded to the treatment groups at 1 h (during MCAo) and then on days 1, 2, 3 and 7 after MCAo. The battery consisted of two tests that have been used previously [35] to evaluate various aspects of neurologic function: (1) the postural reflex test, to examine upper body posture while the animal is suspended by the tail, and (2) the forelimb placing test, to examine sensorimotor integration in forelimb placing responses to visual, tactile and proprioceptive stimuli. Neurological function was graded on a scale of 0–12 (normal score = 0, maximal score = 12) [35]. Only those animals with a high-grade neurological deficit (10 or greater) were used.

### Experimental protocols

DHA (5 mg/kg) or NPD1 (300 µg/kg) was dissolved in saline and administered intravenously at a constant rate over 3 min using an infusion pump at 1 h after 2 h of MCAo. Vehicle-treated rats received an intravenous infusion of a comparable volume of 0.9% sodium chloride.

In series 1 (Behavioral and Immunohistohemistry), DHA or saline treatment (n = 8 in each group); behavioral tests were conducted on days 1, 2, 3 and 7 after MCAo; infarct volume was measured on day 7; GFAP, ED-1, and NeuN immunostaining was performed at 4 and 24 h, and on day 7 (n = 6–8 in each group).

In series 2 (Proteomic study in young animals), DHA or saline treatment; Western blot/immunohistochemical analysis of pAKT, pGSK, and pS6 was conducted in brains at 4 and 24 h (n = 8–10 in each group).

In series 3 (Behavioral study in aged rats), DHA (n = 9), NPD1 (n = 8) or saline (n-10) treatment; behavioral function was evaluated on days 1, 2, 3 and 7 after MCAo.

In series 4 (Lipidomic study in aged rats), DHA or saline treatment (n = 6 in each group); NPD1 content was quantified at 4 h after reperfusion.

In series 5 (Proteomic study in aged animals), DHA, NPD1 or saline treatment (n = 6–8 in each group); Western blot analysis of AKT cell survival signaling was conducted at 4 h after reperfusion.

### Western blot analysis

Animals were sacrificed at 4 or 24 h via an ice-cold saline perfusion. The brain was dissected into two coronal layers and each was divided into left and right hemispheres. Each hemisphere was then sectioned into three penumbral and one core region for a total of 16 sections followed by immediate freezing using liquid N2 to prevent protein degradation. Brain tissue was lysed in RIPA buffer (Sigma Cat#R0278), protease inhibitor cocktail (Sigma Cat#P8340), and phosphatase inhibitor cocktail (Sigma Cat#P2850). Sonicated protein samples were separated on NuPAGE 4–12% Bis-Tris Gels and transferred to nitrocellulose filters. The blots were incubated with primary phosphorylated antibody overnight: pAKT (thr308, ser473) and pGSK (ser9) (Cell Signaling) and pS6 (ser235) (Millipore). Membranes were washed three times with Tris-buffered saline containing 0.1% Tween 20, incubated with anti-rabbit (Cell Signaling) or anti-mouse (Santa Cruz) antibody conjugated with horseradish peroxidase for 1 h, washed three times with Tris-buffered saline containing 0.1% Tween 20, and developed with the enhanced chemiluminescence detection system (Fujifilm image reader LAS -3000). The same blot was stripped (Thermo Scientific cat#21059) and re-probed with total AKT antibody (Santa Cruz) in the manner described above to determine the ratio of phosphorylated to total protein expression. Anti-ACTIN (Sigma) antibody was used as an internal control.

### Histopathology

Animals were perfused with 4% paraformaldehyde on day 7, and brains were removed and embedded in a gelatin matrix using MultiBrain™ Technology (NeuroScience Associates) as previously described [36]. Coronal sections were stained with thionine (Nissl), digitized at nine standardized coronal levels, and the area of infarction was measured and analyzed using MCID™ Core imaging software (Linton) as previously described [35]. An investigator blinded to the experimental groups then outlined the zones of infarction (which were clearly demarcated) as well as the left and right hemispheres of each section. Infarct volume was calculated as the integrated product of cross-sectional area and inter-section distance and corrected for brain swelling.

### Immunostaining

Rats were anesthetized and transcardially perfused with 0.9% saline followed by 4% paraformaldehyde 4 h, 24 h or 7 days after MCAo. Immunohistochemical procedures were performed on the adjacent sections to identify specific vascular and neuronal elements in the ischemic core and penumbra. The following antibodies were used: glial fibrillary acid protein (GFAP, Santa Cruz, SDS Biosciences) to label reactive astrocytes, Cd68/ED-1 (Serotec) for activated microglia/microphages, Neuron-Specific Nuclear Protein (NeuN, Chemicon/Millipore) for neurons, and phosphorylated Protein Kinase B (Ak Mouse Strain Transforming Protein, pAKT 308, Cell Signaling). The number of GFAP, ED-1, NeuN, and pAKT 308 positive cells was counted (Zeiss Axio Imager 4.6.3) in the infarct core and penumbra at the level of the central lesion (bregma level +1.2 mm; magnification 40×). Data were expressed as numbers of positive cells per high-power microscopic field. Brain sections were imaged on a Zeiss LSM-510 Meta laser confocal microscope with a 10× objective (Zeiss Plan-NEOFLUAR 10×/0.3). Bright field imaging was used for pAKT 308 staining. Fluorophore visualization (excitation/emission capture) was achieved as follows: GFAP: DyLight 488 (488 nm/505–530 nm, green) and ED1: DyLight 594 (594 nm/603–636 nm, red). The image resolution was set to 2.26 µm/pixel and the cubic voxel dimension was 129.5 µm. Computer-generated MosaiX processed images of GFAP, ED-1 and GFAP/ED-1 double staining from vehicle and DHA rats were generated.

### Lipidomic analysis

Rats were perfused with cold ice saline 4 h after MCAo onset. For each animal, the brain was removed and blocked into two segments that included the bregma levels +2.7 and −0.3 mm. Next, coronal blocks were divided into right and left hemispheres and cut into two regions (one for the cortex and one for the striatum). NPD1, the DHA-derived stereoselective lipid mediator, was isolated from the penumbra as previously described [7].

### Statistical analysis

Data are presented as mean values ± SEM. Repeated measures analysis of variance (ANOVA) followed by Bonferroni procedures to correct for multiple comparisons were used for intergroup comparisons of neurobehavioral scores over time and infarct areas across coronal levels. Two-tailed Student's *t* tests were used for two-group comparisons. Differences at *P*<0.05 were considered statistically significant.
